# Circulating tumor DNA in molecular assessment feasibly predicts early progression of pancreatic cancer that cannot be identified via initial imaging

**DOI:** 10.1038/s41598-023-31051-7

**Published:** 2023-03-23

**Authors:** Fumiaki Watanabe, Koichi Suzuki, Hidetoshi Aizawa, Yuhei Endo, Yuji Takayama, Nao Kakizawa, Takaharu Kato, Hiroshi Noda, Toshiki Rikiyama

**Affiliations:** Department of Surgery, Saitama Medical Center, Jichi Medical University, 1-847, Amanuma-cho, Omiya-ku, Saitama, 330-8503 Japan

**Keywords:** Pancreatic cancer, Diagnostic markers

## Abstract

Molecular assessment using circulating tumor DNA (ctDNA) has not been well-defined. We recruited 61 pancreatic cancer (PC) patients who underwent initial computed tomography (CT) imaging study during first-line chemotherapy. Initial molecular assessment was performed using droplet digital PCR and defined as the change in *KRAS*-mutated ctDNA before and after treatments, which was classified into five categories: mNT, molecular negative; mCR, complete response; mPR, partial response; mSD, stable disease; mPD, progressive disease. Of 61 patients, 14 diagnosed with PD after initial CT imaging showed significantly worse therapeutic outcomes than 47 patients with disease control. In these 47 patients, initial molecular assessment exhibited significant differences in therapeutic outcomes between patients with and without ctDNA (mPD + mSD vs. mCR + mNT; 13.2 M vs. 21.7 M, *P* = 0.0029) but no difference between those with mPD and mSD + mCR + mNT, suggesting that the presence of ctDNA had more impact on the therapeutic outcomes than change in its number. Multivariate analysis revealed that it was the only independent prognostic factor (*P* = 0.0405). The presence of ctDNA in initial molecular assessment predicted early tumor progression and identified PC patients more likely to benefit from chemotherapy.

## Introduction

Pancreatic ductal adenocarcinoma (PDAC) is a lethal malignancy and has the highest mortality rate among all cancers^1^. The 5-year survival rate in patients with PDAC remains as low as 6% in the USA^2^. Surgery remains the only potentially curative treatment for patients with PDAC^3^. Owing to the propensity of PDAC cells to metastasize early, up to 20% of PDAC patients are eligible for initial resection^2^. Even after curative resection, most patients experience recurrence within a year. Treatment without surgery results in unsatisfactory outcomes and poor prognosis with a median survival of 5–9 months^4^, as observed in patients with unresectable tumors.

Recent improvements in chemotherapy for patients with unresectable PDAC have prolonged survival. The most effective treatment should be determined considering the balance between the patients’ survival benefit and adverse events. Owing to the aggressive nature of the disease, regular monitoring of patients undergoing PDAC treatment is performed using clinical assessments with radiographic imaging studies and several biomarkers in blood to determine response to treatment. Response Evaluation Criteria in Solid Tumors (RECIST) is widely used for the assessment of drug response using radiographic imaging studies. However, defining radiographic responses to chemotherapy and radiation in a rigorous manner remains a challenge^5^. Computed tomography (CT) imaging is widely used but has limited success in accurately assessing disease burden because PDAC is characterized by infiltrating, relatively hypo vascular tumors. Alterations in tumor size and attenuation estimated via CT typically have low accuracy in terms of monitoring tumor response to treatment. Morphological criteria including tumor size, attenuation, and contact with the vessels have been proposed to help assess drug response^6,7^, but tumor size can be overestimated on CT owing to treatment-related changes, such as necrosis and edema, and the change in tumor size has no significant correlation with tumor-free resection margin^7,8^. Similarly, change in tumor attenuation is of limited value for predicting resectability, owing to the challenges in distinguishing necrosis, fibro-inflammation, or edema from residual tumor tissues^9^. Furthermore, changes in tumor size on diagnostic imaging using RECIST cannot be used to reliably predict outcomes^10^. Reliable measurements are required to assess early changes in tumors that may help distinguish responders from non-responders during early periods of the treatment for both minimizing toxicities from ineffective treatment and allowing early adequate adaptation of treatment in non-responders.

Among blood-based biomarkers, serum carbohydrate antigen 19-9 (CA19-9) is the most appropriate biomarker for the management of PDAC. Nevertheless, increased levels of CA19-9 are observed in many benign illnesses, such as liver disease, cholangitis, and pancreatitis, and are not applicable for patients with the Lewis antigen-negative blood group^11^. Additionally, hepatic and pancreatic cysts may also interfere with CA19-9 levels^12,13^. As an alternative to CA19-9, liquid biopsy to track circulating proteins, RNA, and DNA has been used for cancer diagnosis and therapeutic stratification^14^. In particular, circulating tumor DNA (ctDNA) detection in the blood of breast, colorectal, and lung cancer patients, among others, has shown clinical relevance for predicting patient relapses^15–19^. In the context of PDAC, the significance of ctDNA and its prognostic and predictive potential has been widely reported in clinical practice. In patients who underwent surgery, Hadano et al.^20^ reported a cumulative rate of 31% ctDNA detection across stages, with a median survival of 13.6 months vs. 27.6 months in patients with detectable vs. no detectable ctDNA, respectively, and a significant association with overall survival (OS) (*P* < 0.0001). In patients who underwent chemotherapy, Tjensvoll et al.^21^ reported that Kaplan–Meier survival analyses indicated that patients with a positive ctDNA status before or after initiation of chemotherapy had shorter progression-free survival (*P* = 0.064 and *P* = 0.071, respectively). Using univariate analysis, Bernard et al.^22^ also reported that the detection of ctDNA at the presentation of unresectable PDAC was associated with a significant deleterious impact on OS and progression free survival (PFS) (*P* < 0.0001 and *P* = 0.018, respectively).

Liquid biopsy may be an ideal alternative to tumor tissue biopsy^23^, eliminating the limitations associated with the use of tissue samples^24^. The most significant advantage of liquid biopsy over conventional tumor biopsies is that it can be performed multiple times, thereby helping monitor changes in the tumor in real time and assessing treatment response. We have reported that the appearance of ctDNA in colorectal cancer is associated with poor prognosis in the unresectable group^25^. Along with changes in CA19-9 and carcinoembryonic antigen levels, ctDNA monitoring helps understand tumor dynamics. However, no definitive assessments for tracking ctDNA exist.

In this study, molecular assessment was defined as change in appearance and mutation allele frequency (MAF) of ctDNA before and after treatment and classified into five categories. The significance of initial molecular assessment using ctDNA was elucidated in connection with therapeutic outcomes. In particular, we assessed pancreatic cancer patients who had undergone first-line chemotherapy and showed stable changes in the initial CT imaging study. We investigated the potential capability of ctDNA tracking in detecting early tumor progression that cannot be identified using CT imaging.

## Results

### Patient characteristics

Characteristics of 61 patients recruited in this study are shown in Table 1 and Supplementary Table S1. This study was conducted as an exploratory study without calculating the sample size for primary endpoints. Of the unresectable pancreatic cancers, 14 were stage III, 25 were stage IV according to the UICC stage, and 22 were recurrent after surgery. The first-line chemotherapy regimens for these unresectable pancreatic cancers included FOLFIRINOX (FFX) in 22 patients and gemcitabine + nab-paclitaxel (GnP) in 39 patients. The median time to initial assessment for drug response was 42 days. At the initial evaluations in 39 patients treated with GnP, one cycle of GnP was administered in 19 patients, two cycles were administered in 15 patients, and three cycles in five patients. However, in 22 patients treated with FFX, one cycle of FFX was administered in 3 patients, two cycles in 17 patients, and four cycles in two patients. The median observation period was 13.2 months. Of these, 44 patients are dead and 17 are alive. Prior to the investigation of *KRAS-*mutated ctDNA in plasma, *KRAS* assessment was performed in tumor tissues of 61 PDAC patients using RASKET with a sensitivity of 1–5% and ddPCR with a sensitivity of 0.01–0.1%. With respect to frequency, G12D, G12V, 12R, Q61H, G12V + G12R, G12D + G12V + 12R, and wild type were detected in 21 (34.4%), 25 (41.0%), 5 (8.2%), 2 (3.3%), 1 (1.6%), 1 (1.6%), and 4 (6.6%) out of 59 samples, respectively. Two patients did not undergo *KRAS* analysis because of insufficient DNA samples.

**Table 1:** Characteristics of patients.

Characteristics	Value (N = 61)
Sex	
Male	29 (48.4%)
Female	32 (51.6%)
Age (median, 68 years)	
> 68 years	30 (49.2%)
≤ 68 years	31 (50.8%)
UICC stage	
Stage III	14 (23.0%)
Stage IV	25 (41.0%)
Recurrence	22 (36.0%)
CA19-9 level before treatment	
≤ 37 U/mL	52 (85.2%)
> 37 U/mL	9 (14.8%)
First-line chemotherapy regimen	
FOLFIRINOX	22 (36.1%)
Gemcitabine plus nab-paclitaxel	39 (62.9%)
The number of cycles to therapeutic evaluation	
Gemcitabine plus nab-paclitaxel	
1 cycle	19 (48.7%)
2 cycles	15 (38.5%)
3 cycles	5 (12.8%)
FOLFIRINOX	
1 cycle	3 (13.6%)
2 cycles	17 (77.3%)
3 cycles	0
4 cycles	2 (9.1%)
* KRAS* status in tumor tissue	
12D	21 (34.4%)
12V	25 (41.0%)
12R	5 (8.2%)
Q61H	2 (3.3%)
12V, 12R	1 (1.6%)
12D, 12V, 12R	1 (1.6%)
Wild	4 (6.6%)
ND	2 (3.3%)

Data are presented as n (%). UICC, Union for International Cancer Control; CA19-9, carbohydrate antigen 19-9; FOLFIRINOX, folinic acid, fluorouracil, irinotecan, and oxaliplatin; ND, not determined.

### The initial assessments for drug response in various studies

Table 2 shows the initial assessments for drug response in various studies, the radiological imaging, tumor markers, and the molecular response. The radiological assessment using CT imaging (RECIST 1.1) identified 2 patients with CR, 7 patients with PR, 34 patients with SD, and 14 patients with PD. There were 4 patients in whom imaging evaluation was difficult. The disease control group (DCG; CR, PR, and SD) diagnosed using CT imaging study included 28 patients treated with GnP and 15 patients with FFX, wherein no significant difference in the number of patients was seen between them (*P* = 0.5911). Considering change in CA19-9 levels as a tumor marker, 30 patients showed more than a 30% decrease in CA19-9 levels, 14 patients exhibited stable CA19-9 levels, and 17 patients displayed more than a 20% increase in CA19-9 levels. The CA19-9 control group (more than 30% decrease and stable) contained 29 patients treated with GnP and 15 patients with FFX, where no significant difference in the number of patients was seen between them (*P* = 0.6054). The ctDNA-based molecular assessments identified 36 patients with mNT, 9 patients with mCR, 1 patient with mPR, 2 patients with mSD, and 2 patients with mPD. The molecular disease control group (mDCG) was defined as patients with mNT, mCR, mPR, and mSD. The mDCG included 30 patients treated with GnP and 18 patients with FFX, where no significant difference in the number of patients was seen between them (*P* = 0.9023). Notably, *KRAS*-mutated ctDNA disappeared after chemotherapy in 9 patients (mCR). The specific values of MAF of ctDNA before and after chemotherapy are shown in Supplementary Table S2. Supplementary Fig. S1 shows the Venn diagram of the three assessment methods, which revealed that 5 patients exhibited progressive disease as determined via all assessments.

**Table 2:** Initial assessments for drug response using various assessments in 61 patients.

Studies	Assessments		Gemcitabine plus nab-paclitaxel N = 39 (%)	Folfirinox N = 22 (%)	All N = 61 (%)
CT imaging	Disease control group (DCG)	Complete response (CR)	2 (5.1)	0	2 (3.3)
		Partial response (PR)	2 (5.1)	5 (22.7)	7 (11.5)
		Stable disease (SD)	27 (69.3)	11 (50.0)	38 (62.2)
	Progressive disease (PD)	Progressive disease (PD)	8 (20.5)	6 (27.3)	14 (23.0)
CA19-9	The CA19-9 control group (CA19-9-DCG)	More than 30%decrease	20 (51.3)	10 (45.5)	30 (49.2)
		Stable disease (SD)	9 (23.1)	5 (22.7)	14 (23.0)
	Progressive disease (PD)	more than 20% increase	10 (25.6)	7 (31.8)	17 (27.8)
ctDNA	The molecular control group (mDCG)	Molecular negative (mNT)	23 (59.0)	13 (59.1)	36 (59.0)
		Molecular complete response (mCR)	6 (15.4)	3 (13.6)	9 (14.8)
		Molecular partial response (mPR)	0	1 (4.5)	1 (1.6)
		Molecular stable disease (mSD)	1 (2.5)	1 (4.5)	2 (3.3)
	Molecular progressive disease (mPD)	Molecular progressive disease (mPD)	9 (23.1)	4 (18.2)	13 (21.3)

Data are presented as n (%). CA19-9, carbohydrate FOLFIRINOX, folinic acid, fluorouracil, irinotecan, and oxaliplatin.

### Therapeutic outcomes based on the initial assessments

**Figure 1:**
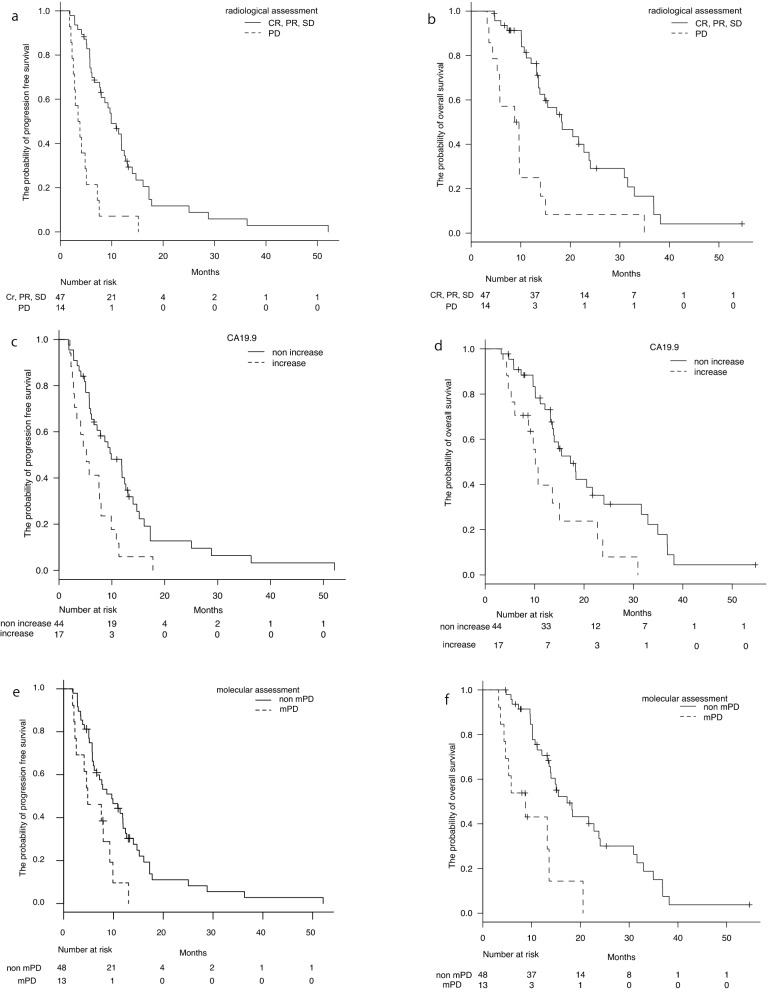
Three assessments of chemotherapy for unresectable pancreatic ductal adenocarcinoma (PDAC) to assess tumor progression and prognosis. (**a**,**c**,**e**) Progression-free survival (PFS) curves based on the changes in *KRAS-*mutated ctDNA and CA19-9 levels, and an initial imaging evaluation of chemotherapy in 61 PDAC patients (*P* = 0.01, 0.00339, and 0.00000365 by log-rank test). (**b**,**d**,**f**) Overall survival curves based on the changes in *KRAS-*mutated ctDNA and CA19-9 levels, and an initial imaging evaluation (*P* = 0.0000654, 0.00659, and 0.000108 by log-rank test). X-axes show months from chemotherapy and Y-axes display the probability of PFS or overall survival.

We compared the therapeutic outcomes of patients in the disease control group and those showing progressive changes according to radiological, tumor markers, and molecular assessments. The radiological assessment showed significantly poor outcomes in the PFS (*P* = 0.00000365) and OS (*P* = 0.000108) of patients with progressive disease (PD) compared with those in the control group (CR + PR + SD, Fig. 1a,b). The tumor marker-based assessment exhibited a poor outcome in the PFS (*P* = 0.00339) and OS (*P* = 0.00659) of patients with a more than 20% increase in CA19-9 levels (CA19-9-PD) compared with those in the control group (more than 30% decrease and stable, Fig. 1c,d). Similarly, the ctDNA-based molecular assessments showed a significantly poor outcome in the PFS (*P* = 0.01) and OS (*P* = 0.0000654) of patients with mPD compared with those of patients in the control group (mNT, mCR, mPR, and mSD, Fig. 1e,f). These data imply that patients showing progressive changes via any assessment showed poor outcomes.

### Impact of molecular assessment in patients in the DCG as determined via CT imaging

We then focused on the 47 patients in the disease control group (CR, PR, and SD) as determined via CT imaging, according to which prognosis was not well distinguished between them (*P* = 0.0776, Supplementary Fig. S2). These patients underwent continuous treatment owing to stable changes even though progressive changes were indicated via other assessments, such as CA19-9 levels or ctDNA. Changes in CA19-9 and ctDNA levels in these 47 patients are shown in Table 3. Ten patients showed a more than 20% increase in CA19-9 levels (CA19-9-PD), of which 6 patients were treated with GnP and 4 patients with FFX. There was no significant difference in the number of patients between treatments (*P* = 0.9426). The molecular assessment identified 6 patients with mPD, of which 4 patients were treated with GnP and 2 patients with FFX. There was no significant difference in the number of patients between treatments (*P* = 1.00).

**Table 3:** Initial assessments for drug response using various assessments in 47 patients diagnosed with disease control during the CT imaging study.

Studies		Assessments	Gemcitabine plus nab-paclitaxel N = 31 (%)	Folfirinox N = 16 (%)	All N = 47 (%)
CA19-9	The CA19-9 control group (CA19-9-DCG)	More than 30% decrease	17 (54.8)	9 (56.3)	26 (55.3)
		Stable	8 (25.8)	3 (18.7)	11 (23.4)
	Progressive disease (CA19-9-PD)	More than 20% increase	6 (19.4)	4 (25.0)	10 (21.3)
ctDNA	The molecular control group (mDCG)	Molecular negative (mNT)	20 (64.5)	10 (62.5)	30 (63.8)
		Molecular complete response (mCR)	6 (19.4)	3 (18.8)	9 (19.1)
		Molecular partial response (mPR)	0	0	0
		Molecular stable disease (mSD)	1 (3.2)	1 (6.2)	2 (4.3)
	Molecular progressive disease (mPD)	Molecular progressive disease (mPD)	4 (12.9)	2 (12.5)	6 (12.8)

Data are presented as n (%). CA19-9, carbohydrate FOLFIRINOX, folinic acid, fluorouracil, irinotecan, and oxaliplatin.

**Figure 2:**
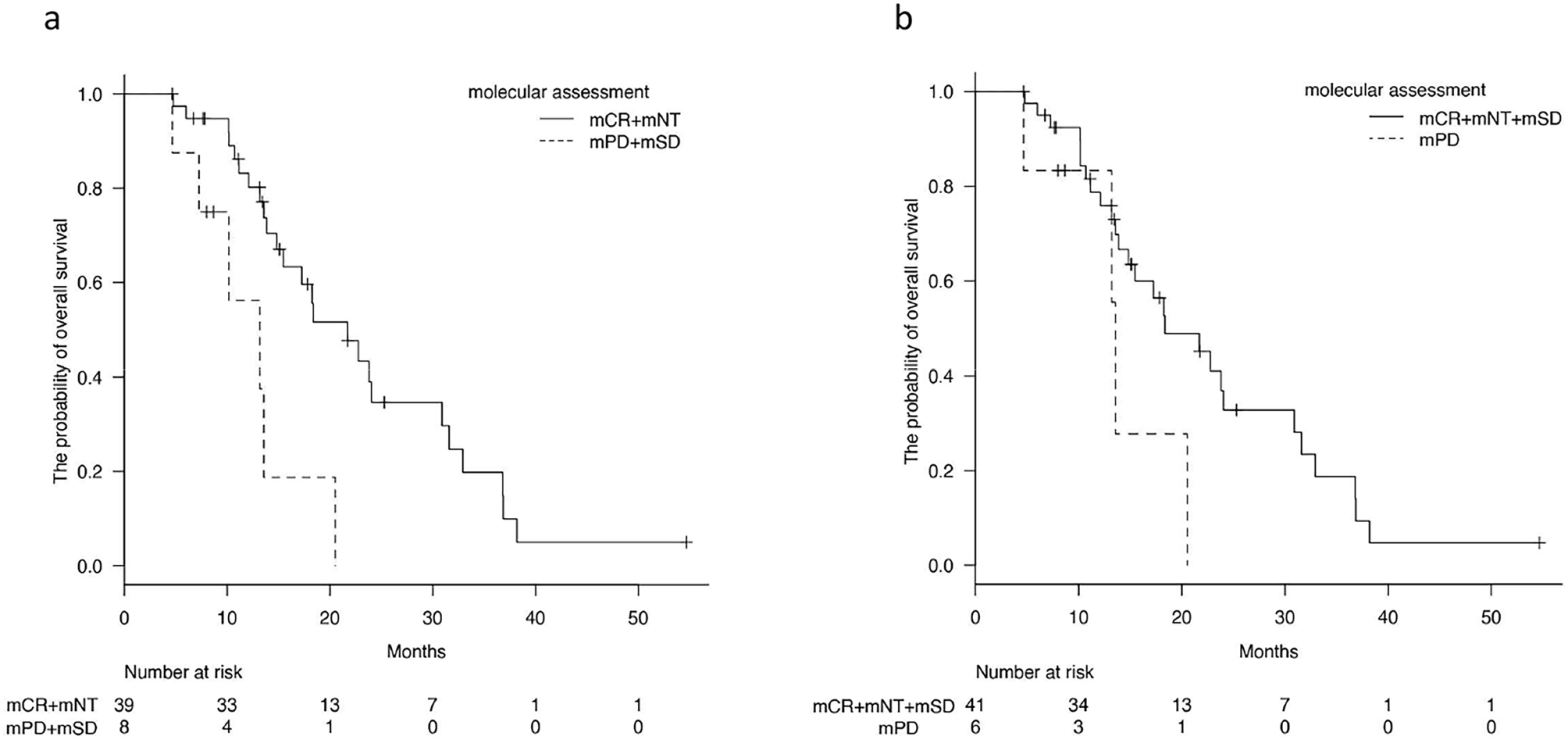
Overall survival (OS) curves based on the molecular assessment in 47 PC patients who were diagnosed with disease control at the initial CT imaging study. (**a**) Comparison of OS between mPD + mSD group and mCR + mNT group (*P* = 0.0194 by log-rank test). (**b**) Comparison of OS between mPD group and mSD + mCR + mNT group (*P* = 0.0756 by log-rank test). X-axis shows months from chemotherapy and Y-axis displays the probability of OS.

We then considered the presence of ctDNA to detect early drug response via molecular assessment and compared treatment outcomes between patients with and without ctDNA (mPD + mSD vs. mCR + mNT). Patients with ctDNA showed significantly poor outcomes as compared to patients without ctDNA in OS (13.2 M in patients with ctDNA vs. 21.7 M in patients without ctDNA, *P* = 0.0029, Fig. 2a) When comparing patients between mPD and mDCG (mSD + mCR + mNT) groups, no significant difference in OS was seen (13.6 M in patients with mPD vs.18.4 M in patients without MCG,* P* = 0.0756, Fig. 2b), suggesting that the presence of ctDNA at the initial molecular assessment had more impact on the therapeutic outcome than change in its number. In addition, the second imaging assessments in 47 corresponding patients demonstrated a significance of the presence of ctDNA at the initial molecular assessment. Seventy-five percent of patients (6/8) with ctDNA after chemotherapy exhibited progression on CT imaging study, while 20% of patients (8/39) without ctDNA showed progression (P = 0.00541, Supplementary Table S3). Similarly, the change in CA19-9 levels showed a significant correlation with therapeutic outcomes, when we compared patients with a more than 30% decrease and stable to those with a more than 20% increase (*P* = 0.0487, Supplementary Fig. S3). To determine significant independent factors in association with clinical outcomes, we performed univariate and multivariate analyses. Table 4 presents the 6 independent demographic and clinicopathological variables used in the univariate analysis for prognosis in the 47 patients in DCG determined using imaging. The tumor marker-based and the molecular assessments were identified as potential prognostic factors (*P* = 0.0487, *P* = 0.0029, respectively; Table 4). The multivariate Cox proportional hazards regression model indicated that the presence of ctDNA (mPD + mSD) determined at initial molecular assessment was the only significant independent factor for prognosis in these patients (Hazard ratio = 2.973, *P* = 0.04056). In all 61 patients, the presence of ctDNA (detectable of ctDNA) also impacted on clinical outcomes including PFS and OS (PFS; *P* = 0.00174 by log-rank test, hazard ratio = 2.68, OS; *P* = 0.0000012 by log-rank test, hazard ratio = 5.22, Supplementary Fig. S4).

**Table 4:** Univariate and multivariate analyses in 47 patients diagnosed with disease control during the CT imaging study.

Prognostic factors	No. of patients	Univariate analysis		Multivariate analysis	
		MST (months)	*P*-value	Hazard ratio (95% CI)	*P*-value
Sex					
Male	22	18.4			
Female	25	18.3	0.691		
Age (median, 68 years)					
≤ 67 years	23	22.8			
> 67 years	24	18.3	0.189		
Unresectable factor					
Stage3 + stage4 (UICC stage)	32	17.3			
Recurrence	15	18.4	0.197		
Chemotherapy					
FOLFIRINOX	16	22.8			
Gemcitabine plus nab-paclitaxel	31	18.3	0.309		
Tumor marker-based assessment (CA-19-9)					
30% decrease	26	18.4		1	Reference
20% increase + stable	21	15.5	0.0487	1.678 (0.7076–3.979)	0.24000
The molecular assessment					
mNT + mCR	39	21.7		1	Reference
mPD + mSD	8	13.2	0.0029	2.973 (1.0480–8.433)	0.04056

MST, median survival time; CI, confidence interval; UICC, Union for International Cancer Control; FOLFIRINOX, folinic acid, fluorouracil, irinotecan, and oxaliplatin; CA19-9, carbohydrate antigen 19-9; mNT, molecular negative; mR, molecular response; mPD, molecular progressive disease.

## Discussion

In this study, we demonstrated the significance of molecular assessment using ctDNA in predicting therapeutic outcomes in patients with unresectable pancreatic cancer who underwent first-line chemotherapy and were diagnosed with disease control during the initial CT imaging study. Patients with mPR, mSD, or mPD had significantly poor therapeutic outcomes, implying that the presence of ctDNA was likely associated with resistance to drug treatments in unresectable pancreatic cancer patients. The detection of ctDNA at initial molecular assessment may be a feature of early tumor progression, which is significantly associated with poor therapeutic outcomes.

A new concept named regression assessment was proposed for the first time by Yin et al.^26^. They combined genomic analysis of resected specimens and liquid biopsy data from 36 PDAC patients who underwent complete resection after neoadjuvant chemotherapy and pathologically diagnosed complete remission (mCR). This was the first study to apply molecular assessment in clinical practice. In their study, three of the six patients with mCR exhibited recurrence compared with six of the 15 non-mCR patients. Seven of the 15 non-mCR patients died during follow-up, whereas only one in six mCR patients died. They concluded that ctDNA existed even in patients with PDAC with pathological complete remission to neoadjuvant chemotherapy, which could possibly predict early recurrence and reduced survival. Del Re et al.^27^ also reported that there was a statistically significant difference in PFS and OS of patients with unresectable PDAC who underwent chemotherapy exhibiting increase vs. stability/reduction in ctDNA in the sample collected at day 15 compared with the sample collected before treatment (median PFS 2.5 vs 7.5 months, *P* = 0.03; median OS 6.5 vs 11.5 months, *P* = 0.009). These results demonstrate that changes in ctDNA are associated with tumor response to chemotherapy, which is consistent with our data; however, this study had some limitations, such as a short observation period and insufficient analysis (no use of multivariate analysis). In our study, the initial molecular assessment was defined using change in appearance and MAF of ctDNA before and after treatment and classified into five categories including mNT, mCR, mPR, mSD, and mPD in 61 patients who underwent first-line chemotherapy. Patients with mPD showed worse therapeutic outcomes than those in the molecular control group (mNT, mCR, mPR, and mSD). Furthermore, the presence of ctDNA was more important in determining drug response in pancreatic cancer patients who were diagnosed with disease control during the initial CT imaging study. As ctDNA has also been reported to be involved in micro metastasis^28^, the disappearance of ctDNA may have a greater impact in improving prognosis in patients who have undergone chemotherapy.

Several studies have suggested the integration of established and experimental protein biomarkers with ctDNA analysis for solid tumors, including pancreatic cancer, for early diagnostics^29,30^, identification of minimal residual disease^31^, and molecular monitoring for advanced disease. Cohen et al.^29^ and Hussung et al.^32^ reported that ctDNA positivity and increase in CA19-9 levels were partially overlapping, and combining both parameters could help identify a larger cohort of patients with poor outcomes. However, their study was limited by the cutoff value of CA19-9 levels, as it was not optimal for predicting the prognosis and recurrence of pancreatic cancer. Therefore, the overlap of ctDNA positivity and increase in CA19-9 levels were partial. However, in our previous studies, we showed that longitudinal monitoring of *KRAS*-mutated ctDNA could help identify tumor dynamics in various treatments and revealed a significant correlation between *KRAS*-mutated ctDNA and CA19-9 levels in pancreatic cancer patients^33,34^. Based on the evidence of this relationship between *KRAS*-mutated ctDNA and CA19-9, we determined 949.7 U/mL as an optimal cut-off level for CA19-9, which was an independent risk factor for recurrence and prognosis in surgical patients^33,34^. In this study, we evaluated changes in CA19-9 levels before and after chemotherapy rather than relying on cutoff values because we think that the dynamic changes along with treatment are more important than cutoff levels. Only five patients (8.2%) were classified as having a worsening disease via all three assessment methods (imaging, CA 19-9, and molecular assessment), indicating that the overlap was partial.

The induction of anticancer agents such as FOLFIRINOX (FFX) and nab-paclitaxel + gemcitabine (GnP) lead to frequent and severe adverse events compared with gemcitabine, which was widely used in the past. Conroy et al.^35^ reported that incidences of grade 3 or 4 neutropenia, febrile neutropenia, thrombocytopenia, diarrhea, and sensory neuropathy were significantly higher in patients treated with FOLFIRINOX (FFX) than in those treated with gemcitabine. Von Hoff et al.^36^ reported that the most frequently reported nonhematologic adverse events related to treatment were fatigue (in 54% of patients), alopecia (in 50%), and nausea (in 49%) in patients treated with nab-paclitaxel + gemcitabine (GnP). Treatment-related adverse events of grade 3 or higher result in a dose reduction or the discontinuation of the treatment. Patients with stable disease as diagnosed by the CT imaging study would undergo continued treatments and showed more or less adverse events. Initial molecular assessment enables avoidance of this continued treatment in patients with ctDNA who are more unlikely to benefit from this continuation. In contrast, patients without ctDNA (CR + NT) are expected to have better therapeutic outcomes (MST; 21.7 months), and the treatments using FFX or GnP should be continued.

Several limitations that were associated with the present study warrant mention. This was a retrospective cohort study conducted at a single institution, and the number of enrolled patients was relatively small. Therefore, further studies are needed to explore the potential of biomarker-based therapeutic interventions for PDAC. A switch or continuation of chemotherapy regimens based on molecular monitoring appears feasible yet requires extensive clinical validation in interventional trials.

In summary, herein, we proposed a clinically feasible approach of definitive molecular assessment using ctDNA of PDAC during chemotherapy. This initial molecular assessment enables the prediction of early tumor progression, which cannot be determined by imaging, and helps identify patients more likely to benefit from chemotherapy in patients with unresectable pancreatic cancer. Although our findings should be interpreted within the study limitations and further examinations are required to draw a definitive conclusion, we believe that our study provides important insight into the appropriate selection of treatment routes for patients with PDAC.

## Methods

### Patients and study design

We prospectively recruited 61 clinically diagnosed patients with unresectable PDAC and performed 1st-line chemotherapy between July 2015 and December 2020, and 122 blood samples before and after chemotherapy were collected from the same patients at Saitama Medical Center, Jichi Medical University, Japan. We evaluated CT images, CA19-9 levels, and *KRAS*-mutated ctDNA, a median of 42 days after the initial induction of first-line chemotherapy. Progression in all patients was determined based on routine clinical evaluation by at least one radiologist and several surgeons based on RECIST 1.1 criteria with only one image evaluation. We defined the disease control group (DCG) including complete response (CR), partial response (PR), stable disease (SD), and progressive disease (PD) using initial CT imaging. All patients provided written informed consent for the examination of their tissue and plasma and the use of their clinical data. The study protocol was approved by the research ethics committee of Jichi Medical University (approval no. R19‐30; Saitama, Japan) and conformed to the ethical guidelines of the World Medical Association Declaration of Helsinki.

### Analysis of *KRAS* status in PDAC tissues

*KRAS* status in PDAC tissues was evaluated with RASKET and droplet digital PCR (ddPCR) using endoscopic ultrasound-guided fine-needle aspiration samples or surgical specimens. *KRAS* status of tumor tissues was analyzed using RASKET by a clinical testing company (Special Reference Laboratories, Tokyo, Japan). Consequently, tissue DNA was extracted from formalin-fixed paraffin-embedded (FFPE) tissues using the QIAamp DNA FFPE Tissue Kit (Qiagen, Hilden, Germany) according to the manufacturer’s instructions. Earlier studies have reported that point mutations at codon 12 of the *KRAS* oncogene primarily include G12V, G12D, and G12R, whereas other types of *KRAS* point mutations are rarely detected in patients with PDAC^37–39^. Therefore, these three types of *KRAS* mutations were predominantly identified via ddPCR. In addition, Q61H and Q61L, other types of *KRAS* mutations that emerged prior to drug resistance, were verified in two patients using ddPCR after initial determination using RASKET. *KRAS* status in two patients could not be assessed because of the unavailability of samples.

## Plasma sample collection and processing

In total, 122 blood samples were collected from patients with unresectable PDAC at the hospital. From each patient, 7 mL of whole blood was drawn into EDTA-containing tubes, and plasma was collected by centrifugation at 3000×*g* for 20 min at 4 °C within approximately 4 h of collection, followed by centrifugation at 16,000×*g* for 10 min at 4 °C in a fresh tube. Plasma samples were separated from peripheral blood cells and stored at − 80 °C until DNA extraction.

### Extraction of circulating cell-free DNA

Circulating cell-free DNA was extracted from 2 mL of plasma using the QIAamp Circulating Nucleic Acid Kit (Qiagen, Hilden, Germany) according to the manufacturer’s instructions.

### ddPCR analyses

*KRAS* status in tumor tissues and plasma was analyzed using the Bio-Rad QX200 ddPCR system (Bio-Rad Laboratories, Hercules, CA, USA), as previously described^25,33,34^. Point mutations identified in tissues were monitored and detected in blood with no additional exploration of point mutations required using ddPCR. The negative threshold of mutant allelic frequency was indicated as  < 5 copies/1 mL plasma, as previously described^34^.

### Response criteria for target lesions by CT imaging (RECIST 1.1 criteria)

Complete Response (CR) was defined as the disappearance of all non-nodal target lesions. Any nodal target lesions must have a reduction in the short axis to < 10 mm. When nodal target lesions were selected at baseline, the sum diameters may not be 0 mm even if the target lesion response was CR.

Partial Response (PR) was defined as at least a 30% decrease in the sum of diameters of target lesions, with the reference being the baseline sum diameters.

Progressive Disease (PD) was defined as at least a 20% increase in the sum of diameters of target lesions, with the reference being the smallest sum diameters in the study (this included the baseline sum if that was the smallest in the study). The sum of diameters must also demonstrate an absolute increase of at least 5 mm.

Stable Disease (SD) was defined as neither sufficient shrinkage to qualify for PR nor sufficient increase to qualify for PD, with the reference being the smallest sum diameters in the study.

### Molecular assessment

We defined molecular assessment as the change in *KRAS*-mutated ctDNA levels before and after treatment (Supplementary Table S2): (1) no appearance of *KRAS*-mutated ctDNA before and after treatment was defined as molecular negative (mNT); (2) disappearance of *KRAS*-mutated ctDNA after chemotherapy was defined as molecular complete response (mCR); (3) 30% decrease in mutant allelic frequency after treatment was defined as molecular partial response (mPR); (4) new appearance of *KRAS*-mutated ctDNA or 20% increase in the MAF of ctDNA after treatment was defined as molecular progressive disease (mPD); (5) neither sufficient decrease to qualify for mPR nor sufficient increase to qualify for mPD was defined as molecular stable disease (mSD).

### Statistical analysis

We measured progression-free survival (PFS) and overall survival (OS) to assess prognosis. PFS was defined as the time from the start of chemotherapy to confirmation of progression based on initial radiological findings. OS was defined as the time from the start of chemotherapy to the occurrence of the event. A Cox proportional hazards regression model was used to evaluate the association between overall mortality and other factors in univariate and multivariate analyses. The following variables were analyzed in patients: sex; age at the start of chemotherapy (≤ 68 years vs. > 68 years); unresectable factor (stage III + stage IV vs. recurrence); chemotherapy (gemcitabine plus nab-paclitaxel vs. FOLFIRINOX); and the change in CA19-9 levels after chemotherapy. PFS and OS curves were constructed using the Kaplan–Meier method. Several factors with a *P*-value of < 0.1 in univariate analysis were subjected to multivariate analysis, and a *P*-value of 0.05 was considered statistically significant. All statistical analyses were performed using EZR version 1.31 (Saitama Medical Center, Jichi Medical University, Saitama, Japan). We also used R version 3.1.1 (The R Foundation for Statistical Computing, Vienna, Austria) as a graphical interface.

## Supplementary Information

Supplementary Legends.

Supplementary Figure S1.

Supplementary Figure S2.

Supplementary Figure S3.

Supplementary Figure S4.

Supplementary Table S1.

Supplementary Table S2.

Supplementary Table S3.

## Data Availability

The datasets that support the findings of this study are available from the corresponding author on reasonable request.
