# Supplementary material for: Circulating tumor DNA in molecular assessment feasibly predicts early progression of pancreatic cancer that cannot be identified via initial imaging
Source: Sci Rep. 2023 Mar 23;13:4809. doi: 10.1038/s41598-023-31051-7 (PMC10036464; doi:10.1038/s41598-023-31051-7)
Supplement: Supplementary file 1 — Supplementary Legends. [file 41598_2023_31051_MOESM1_ESM.docx]

Circulating tumor DNA in molecular assessment feasibly predicts early progression of pancreatic cancer that cannot be identified via initial imaging

Fumiaki Watanabe^1^, Koichi Suzuki^1^, Hidetoshi Aizawa^1^, Yuhei Endo^1^, Yuji Takayama^1^, Nao Kakizawa^1^, Takaharu Kato^1^, Hiroshi Noda^1^, Toshiki Rikiyama^1^

**Supporting information**

**Supplementary Figure S1.** Venn diagram of progressive disease as determined via three assessments in 61 patients with unresectable pancreatic ductal adenocarcinoma (PDAC).

**Supplementary Figure S2.** Overall survival (OS) curves based on an initial image evaluation after chemotherapy in patients with unresectable pancreatic ductal adenocarcinoma (PDAC) excluding radiological progressive disease. The median overall survival time was 24.03 months in complete response, 13.87 months in partial response, and 20.53 months in stable disease.

**Supplementary Figure S3.** Overall survival (OS) curves based on the change in carbohydrate antigen 19-9 (CA19-9) levels after chemotherapy in patients with unresectable pancreatic ductal adenocarcinoma (PDAC) excluding radiological progressive disease. The median overall survival time was 18.4 months in the decrease group of CA19-9 and 15.5 months in the non-decrease group (*P* = 0.0487 using log-rank test).

**Supplementary Figure S4.** (a) Progression-free survival curves comparing patients with and without *KRAS-*mutated ctDNA in 61 PDAC patients (*P* = 0.00174 by log-rank test, hazard ratio = 2.68). (b) Overall survival curves comparing patients with and without *KRAS-*mutated ctDNA in 61 PDAC patients (*P* = 0.0000012 by log-rank test, hazard ratio = 5.22).
