# Supplementary figures and images for: Circulating tumor DNA in molecular assessment feasibly predicts early progression of pancreatic cancer that cannot be identified via initial imaging
Source: Sci Rep. 2023 Mar 23;13:4809. doi: 10.1038/s41598-023-31051-7 (PMC10036464; doi:10.1038/s41598-023-31051-7)

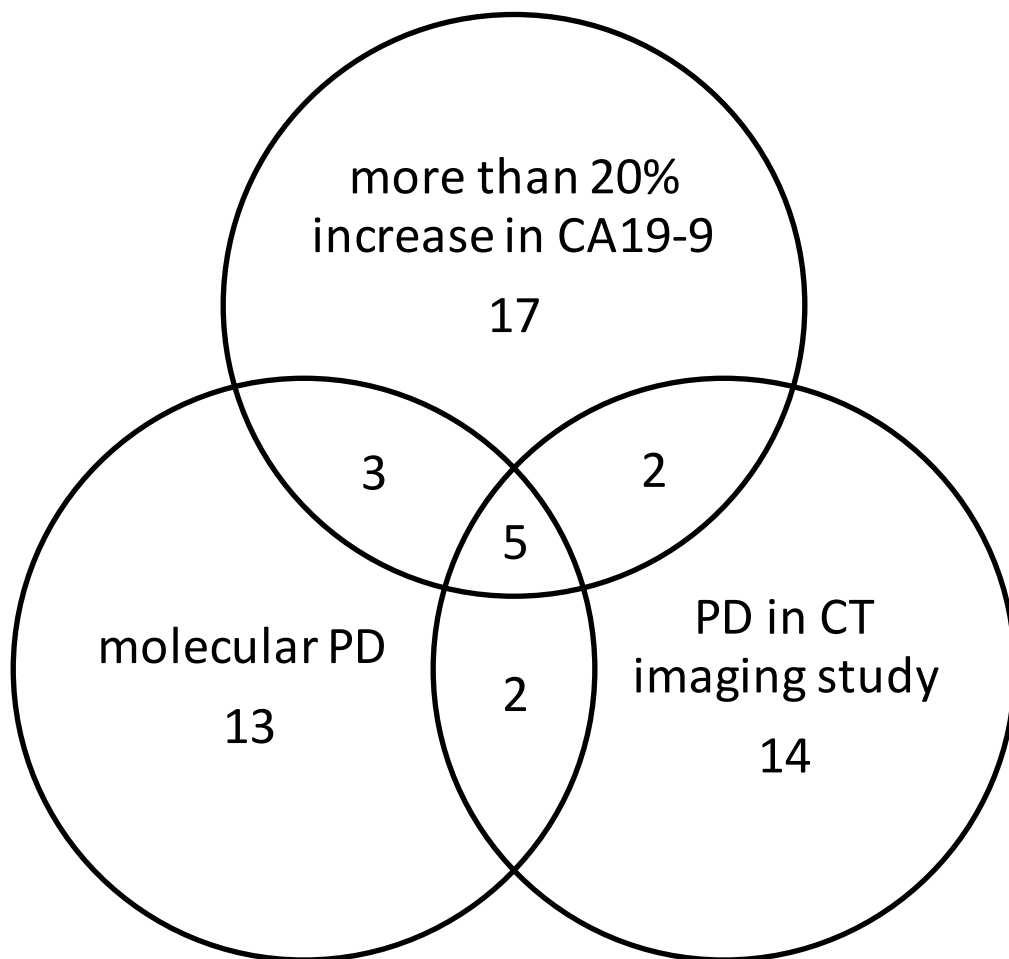

Supplement: Supplementary file 2 — Supplementary Figure S1. [file 41598_2023_31051_MOESM2_ESM.pdf]

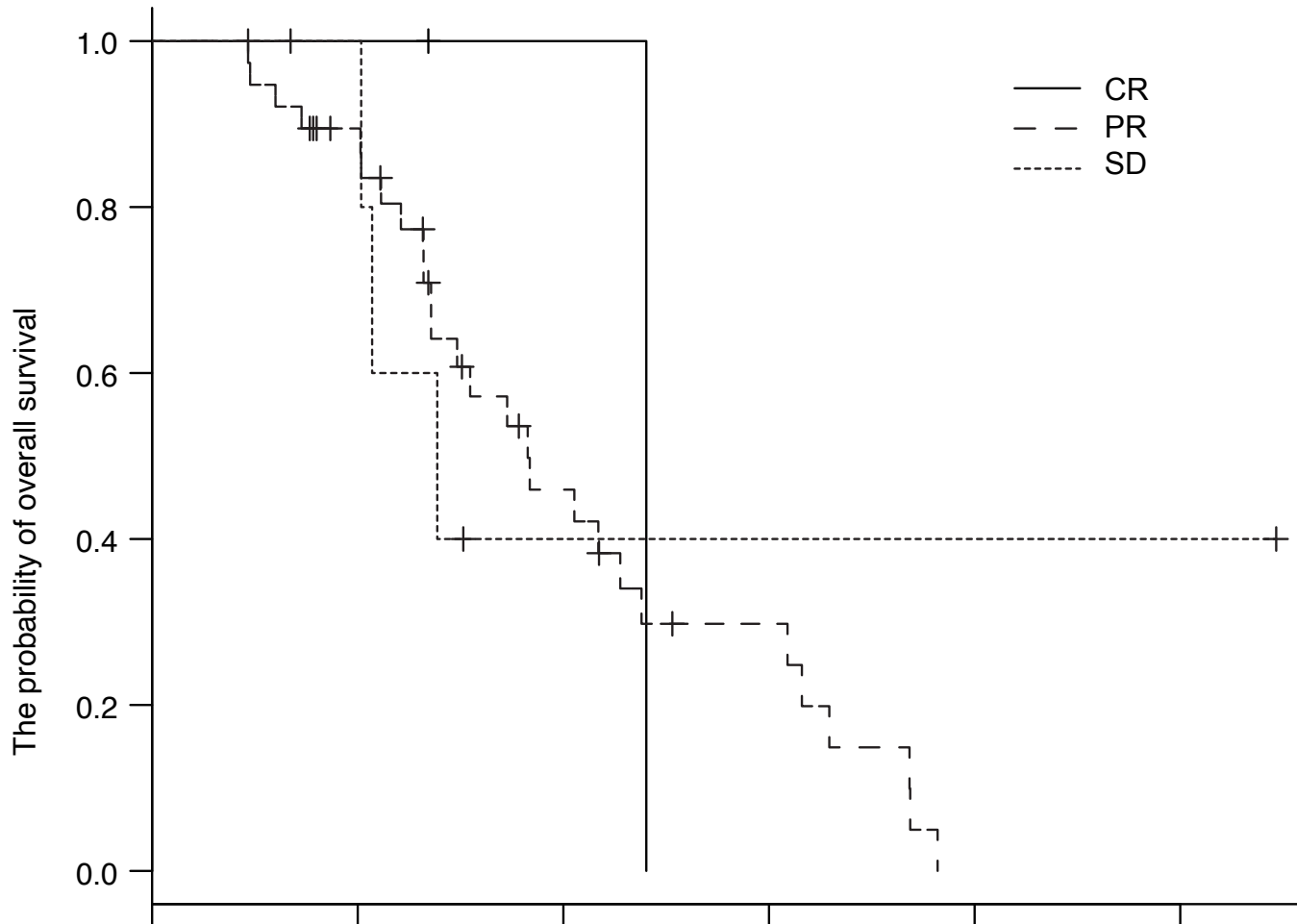

Months

Number at risk

|    |    |    |    |   |   |   |
|----|----|----|----|---|---|---|
| CR | 2  | 2  | 1  | 0 | 0 | 0 |
| PR | 7  | 5  | 1  | 1 | 1 | 1 |
| SD | 38 | 30 | 12 | 6 | 0 | 0 |

Supplement: Supplementary file 3 — Supplementary Figure S2. [file 41598_2023_31051_MOESM3_ESM.pdf]

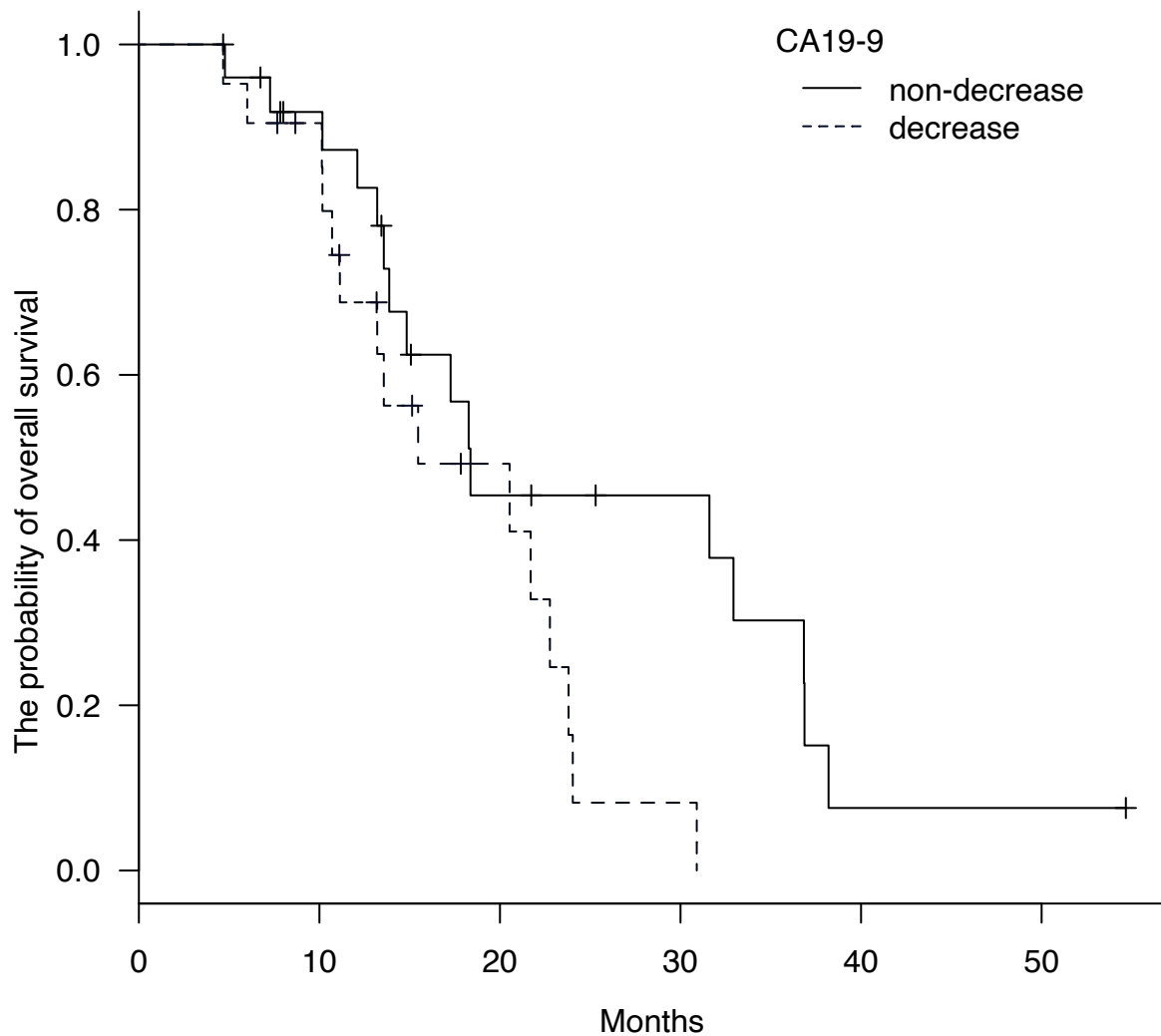

Number at risk

non-decrease 26  
decrease 21

20  
17

8  
6

6  
1

1  
0

1  
0

Supplement: Supplementary file 4 — Supplementary Figure S3. [file 41598_2023_31051_MOESM4_ESM.pdf]

a

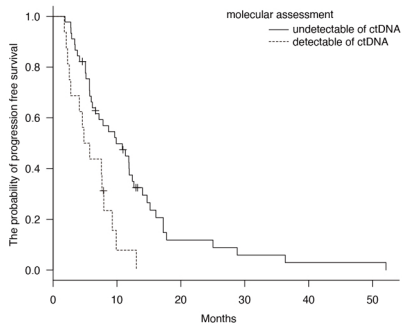

b

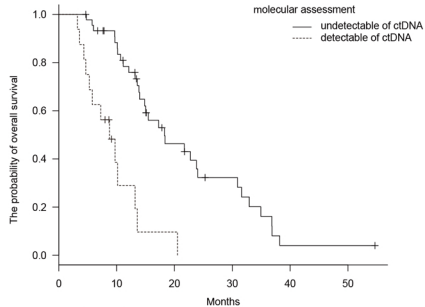

Supplement: Supplementary file 5 — Supplementary Figure S4. [file 41598_2023_31051_MOESM5_ESM.pdf]
