# Supplementary material for: Circulating tumor DNA in molecular assessment feasibly predicts early progression of pancreatic cancer that cannot be identified via initial imaging
Source: Sci Rep. 2023 Mar 23;13:4809. doi: 10.1038/s41598-023-31051-7 (PMC10036464; doi:10.1038/s41598-023-31051-7)
Supplement: Supplementary file 6 — Supplementary Table S1. [file 41598_2023_31051_MOESM6_ESM.docx]

Supplementary Table S1. Clinical information of patients with unresectable pancreatic cancer in connection with molecular findings

| Patients | Sex | Age (years) | Unresectable factor | *KRAS* mutation in tissue | CA19-9 level before chemotherapy | CA19-9 level after chemotherapy | Mutant allelic frequency before chemotherapy (copies/1mLplasma) | Mutant allelic frequency after chemotherapy　(copies/1mLplasma) | Molecular findings | Radiological findings | Chemotherapy | Cycles to effect assessment | Outcome |
| --- | --- | --- | --- | --- | --- | --- | --- | --- | --- | --- | --- | --- | --- |
| 1 | f | 75 | Recurrence (liver) | 12D | 123.5 | 122.8 | <5.0 | 6.4 | mPD | SD | GnP | 1 | death |
| 2 | f | 75 | Liver metastasis | Q61H | 22.7 | 9.1 | 20 | <5.0 | mCR | SD | GnP | 2 | death |
| 3 | m | 72 | Locally advanced | NA | 576 | 1816 | <5.0 | <5.0 | mNT | PR | GnP | 1 | death |
| 4 | m | 56 | Locally advanced | 12V | 393.2 | 196.7 | <5.0 | <5.0 | mNT | SD | FOLFIRINOX | 1 | death |
| 5 | f | 68 | Recurrence (pancreas) | 12V | 79.8 | 15.3 | <5.0 | <5.0 | mNT | SD | GnP | 2 | death |
| 6 | m | 49 | Recurrence (pancreas, liver) | 12D | 435.2 | 218.3 | <5.0 | <5.0 | mNT | SD | FOLFIRINOX | 1 | death |
| 7 | f | 44 | Recurrence (local, liver) | 12D | 1422.3 | 2872.7 | 36 | 162 | mPD | PD | FOLFIRINOX | 2 | death |
| 8 | m | 70 | Recurrence (lymph node) | 12D | 466.8 | 215.3 | 32 | 26.2 | mSD | SD | GnP | 3 | death |
| 9 | f | 65 | Locally advanced | 12V | 132 | 72.5 | 7.6 | <5.0 | mCR | SD | GnP | 2 | death |
| 10 | f | 74 | Recurrence (local) | 12D | 86.9 | 99.7 | <5.0 | <5.0 | mNT | CR | GnP | 1 | death |
| 11 | f | 68 | Recurrence (Peritoneal dissemination) | 12V | 209.9 | 2031.8 | <5.0 | <5.0 | mNT | SD | GnP | 1 | death |
| 12 | f | 66 | Recurrence (liver) | 12V | 153.5 | 69.4 | <5.0 | <5.0 | mNT | PD | GnP | 1 | death |
| 13 | m | 69 | Peritoneal dissemination, liver, lung metastasis | 12V | 326645.6 | 281508.7 | 150 | 2852 | mPD | PD | GnP | 2 | death |
| 14 | f | 77 | Recurrence (local) | 12D | 175.1 | 164.4 | <5.0 | 15.2 | mPD | SD | GnP | 1 | death |
| 15 | m | 78 | Recurrence (Peritoneum) | 12V | 3356.8 | 621.1 | <5.0 | <5.0 | mNT | SD | GnP | 1 | death |
| 16 | f | 73 | Recurrence (local) | 12V | 113.1 | 43 | <5.0 | <5.0 | mNT | SD | GnP | 2 | death |
| 17 | f | 41 | Liver, lung metastasis | 12D | 15276.9 | 38833.8 | <5.0 | 14.5 | mPD | SD | FOLFIRINOX | 4 | death |
| 18 | m | 71 | Recurrence (lymph node) | 12D | 340.4 | 216.9 | <5.0 | <5.0 | mNT | SD | GnP | 3 | death |
| 19 | f | 66 | Lymph node metastasis | 12V | 11.6 | 8 | 32.4 | <5.0 | mCR | PR | FOLFIRINOX | 2 | alive |
| 20 | f | 70 | Recurrence (lymph node, peritoneum) | 12D | 344.5 | 177.3 | <5.0 | <5.0 | mNT | PD | GnP | 2 | death |
| 21 | m | 74 | Recurrence (lung, peritoneum) | 12V | 350.8 | 2289.9 | <5.0 | 6.0 | mPD | PD | GnP | 1 | death |
| 22 | m | 69 | Liver metastasis | 12V | 2143.2 | 665.9 | <5.0 | <5.0 | mNT | PD | GnP | 2 | death |
| 23 | m | 73 | Recurrence (lung) | 12V | 8.4 | 10.2 | <5.0 | 7.72 | mPD | PD | GnP | 1 | alive |
| 24 | f | 45 | Locally advanced | 12D | 174.9 | 124.6 | <5.0 | <5.0 | mNT | PD | FOLFIRINOX | 2 | death |
| 25 | f | 76 | Lymph node metastasis | 12R | 15.1 | 16.1 | <5.0 | <5.0 | mNT | SD | GnP | 1 | death |
| 26 | f | 69 | Recurrence (local) | Wild | 9.2 | 12.3 | <5.0 | <5.0 | mNT | SD | GnP | 1 | death |
| 27 | f | 82 | Lymph node metastasis | 12V | 423.3 | 56.4 | <5.0 | <5.0 | mNT | SD | GnP | 2 | death |
| 28 | m | 66 | Peritoneal dissemination | 12V | 401.3 | 473.8 | 29.6 | <5.0 | mCR | SD | FOLFIRINOX | 2 | death |
| 29 | f | 56 | Liver metastasis | 12D | 14085.2 | 49980.3 | <5.0 | <5.0 | mNT | PD | FOLFIRINOX | 2 | death |
| 30 | f | 58 | Recurrence (liver, peritoneum) | 12V | 10.1 | 19.8 | <5.0 | <5.0 | mNT | SD | FOLFIRINOX | 2 | death |
| 31 | f | 62 | Liver, lymph node metastasis | 12V | 23440.4 | 95477.8 | 14.4 | 15.4 | mSD | PR | FOLFIRINOX | 1 | death |
| 32 | f | 66 | Recurrence (liver) | 12D | 50.8 | 293 | 15 | 678 | mPD | PD | GnP | 2 | death |
| 33 | m | 68 | Locally advanced | 12V | 340.3 | 147.7 | <5.0 | <5.0 | mNT | SD | GnP | 1 | death |
| 34 | f | 73 | Liver, lymph node metastasis | Wild | 372 | 790.8 | <5.0 | 9.2 | mPD | SD | GnP | 1 | death |
| 35 | f | 63 | Locally advanced | 12D | 1582.7 | 849 | <5.0 | <5.0 | mNT | SD | FOLFIRINOX | 2 | death |
| 36 | m | 41 | Recurrence (Peritoneum) | 12R | 79.6 | 162.9 | 14.6 | <5.0 | mCR | SD | FOLFIRINOX | 2 | death |
| 37 | m | 55 | Liver metastasis | 12D | 4029.4 | 1988.1 | <5.0 | <5.0 | mNT | PR | FOLFIRINOX | 2 | death |
| 38 | m | 66 | Liver metastasis | 12D | 28550 | 14755.1 | 14 | <5.0 | mCR | SD | GnP | 2 | death |
| 39 | m | 59 | Lymph node metastasis | 12R | 943.9 | 425.7 | <5.0 | <5.0 | mNT | PD | FOLFIRINOX | 2 | death |
| 40 | m | 50 | Recurrence (liver) | 12R | 1.2 | 2.6 | 28.8 | 19.8 | mPR | PD | FOLFIRINOX | 2 | death |
| 41 | f | 69 | Lymph node metastasis | 12V | 1447.1 | 1454.3 | 8.52 | <5.0 | mCR | SD | GnP | 2 | death |
| 42 | m | 77 | Locally advanced | 12D | 631.1 | 309.9 | <5.0 | <5.0 | mNT | SD | GnP | 1 | alive |
| 43 | m | 66 | Recurrence (liver) | 12V | 574.3 | 258.8 | <5.0 | <5.0 | mNT | CR | GnP | 1 | alive |
| 44 | f | 43 | Liver metastasis | 12D | 34047.8 | 33660.3 | 35.2 | 192 | mPD | PD | FOLFIRINOX | 2 | death |
| 45 | f | 70 | Liver, peritoneal, metastasis | Wild | 41426.8 | 25400.3 | 144 | <5.0 | mCR | SD | GnP | 2 | death |
| 46 | m | 77 | Liver metastasis | 12D | 386.5 | 368.9 | <5.0 | <5.0 | mNT | PR | GnP | 3 | alive |
| 47 | f | 72 | Peritoneal dissemination | NA | 21.4 | 13.5 | <5.0 | <5.0 | mNT | SD | GnP | 1 | alive |
| 48 | f | 75 | Liver metastasis | Q61H | 182.4 | 663.9 | 45.4 | <5.0 | mCR | SD | GnP | 1 | alive |
| 49 | m | 79 | Recurrence (peritoneum) | 12D | 38.1 | 20.1 | <5.0 | <5.0 | mNT | SD | GnP | 2 | alive |
| 50 | m | 82 | Lung metastasis | 12D | 3003.7 | 2148.2 | <5.0 | <5.0 | mNT | SD | GnP | 1 | death |
| 51 | m | 67 | Locally advanced | 12D | 1899.3 | 2072.2 | <5.0 | <5.0 | mNT | SD | FOLFIRINOX | 2 | alive |
| 52 | m | 70 | Locally advanced | 12V | 308 | 101.6 | <5.0 | <5.0 | mNT | SD | FOLFIRINOX | 2 | alive |
| 53 | f | 61 | Locally advanced | 12V | 1451.5 | 258 | <5.0 | <5.0 | mNT | SD | GnP | 3 | death |
| 54 | f | 60 | Locally advanced | 12V | 1160.2 | 119 | <5.0 | <5.0 | mNT | SD | GnP | 3 | alive |
| 55 | f | 59 | Locally advanced | 12R | 2252.3 | 1830.9 | <5.0 | <5.0 | mNT | SD | FOLFIRINOX | 2 | alive |
| 56 | m | 73 | Bone metastasis | 12D | 214.9 | 213.1 | <5.0 | <5.0 | mNT | SD | GnP | 2 | alive |
| 57 | f | 74 | Lymph node metastasis | 12V | 31 | 141.7 | 12.6 | 32.2 | mPD | PD | GnP | 2 | death |
| 58 | m | 75 | Liver metastasis | 12V | 41193.9 | 65480.5 | 216 | 468 | mPD | SD | GnP | 1 | alive |
| 59 | f | 45 | Locally advanced | 12V | 361.4 | 183.1 | <5.0 | 13 | mPD | SD | FOLFIRINOX | 2 | alive |
| 60 | m | 65 | Liver metastasis | Wild | 217.4 | 132.5 | <5.0 | <5.0 | mNT | PR | FOLFIRINOX | 2 | alive |
| 61 | m | 62 | Locally advanced | 12V | 1737.1 | 719.9 | <5.0 | <5.0 | mNT | PR | FOLFIRINOX | 4 | alive |

CA19-9, carbohydrate antigen 19-9; NA, not applicable; mNT, molecular negative; mCR, molecular complete response; mR, molecular response; mSD, molecular stable disease; mPD, molecular progressive disease; CR, complete response; PR, partial response; SD, stable disease; PD, progressive disease; NE, not evaluation; FOLFIRINOX, folinic acid + fluorouracil + irinotecan + oxaliplatin; GnP, gemcitabine plus nab-paclitaxel.
