# Supplementary material for: Circulating tumor DNA in molecular assessment feasibly predicts early progression of pancreatic cancer that cannot be identified via initial imaging
Source: Sci Rep. 2023 Mar 23;13:4809. doi: 10.1038/s41598-023-31051-7 (PMC10036464; doi:10.1038/s41598-023-31051-7)
Supplement: Supplementary file 7 — Supplementary Table S2. [file 41598_2023_31051_MOESM7_ESM.docx]

Supplemental Table S2. Definition of molecular assessments

|  | Before chemotherapy | After chemotherapy |  |
| --- | --- | --- | --- |
| Molecular negative (mNT) | No appearance of *KRAS*-mutated ctDNA | No appearance of *KRAS*-mutated ctDNA |  |
| Molecular complete response (mCR) | Presence of *KRAS*-mutated ctDNA | Disappearance of *KRAS*-mutated ctDNA |  |
| Molecular partial response (mPR) | Presence of *KRAS*-mutated ctDNA | Presence of *KRAS*-mutated ctDNA | 30% decrease in molecular allelic frequency |
| Molecular stable disease (mSD) | Presence of *KRAS*-mutated ctDNA | Presence of *KRAS*-mutated ctDNA | Neither sufficient decrease to qualify for mPR nor sufficient increase to qualify for mPD |
| Molecular progressive disease (mPD) | No appearance of *KRAS*-mutated ctDNA | Presence of *KRAS*-mutated ctDNA |  |
|  | Presence of *KRAS*-mutated ctDNA | Presence of *KRAS*-mutated ctDNA | 20% increase in molecular allelic frequency |

ctDNA, circulating tumor DNA.
