# Supplementary material for: Circulating tumor DNA in molecular assessment feasibly predicts early progression of pancreatic cancer that cannot be identified via initial imaging
Source: Sci Rep. 2023 Mar 23;13:4809. doi: 10.1038/s41598-023-31051-7 (PMC10036464; doi:10.1038/s41598-023-31051-7)
Supplement: Supplementary file 8 — Supplementary Table S3. [file 41598_2023_31051_MOESM8_ESM.docx]

**Supplementary Table S3**. Second radiological assessments after chemotherapy in 47 patients diagnosed with disease control during the initial CT imaging study

| **Studies** | | **Second radiological assessments after chemotherapy** | | **ALL** N=47 |
| --- | --- | --- | --- | --- |
| **ctDNA assessments** | | Non-PD | PD |  |
| Undetectable of ctDNA after chemotherapy | Molecular negative (mNT) | 31 | 8 | 39 (83.0) |
|  | Molecular complete response (mCR) |  |  |  |
| Detactable of ctDNA after chemotherapy | Molecular partial response (mPR) | 2 | 6 | 8 (17.0) |
|  | Molecular stable disease (mSD) |  |  |  |
|  | Molecular progressive disease (mPD) |  |  |  |

Data are presented as n (%). PD, progressive disease.
